# Immunomodulatory Effects of Liriope Platyphylla Water Extract on Lipopolysaccharide-Activated Mouse Macrophage

**DOI:** 10.3390/nu4121887

**Published:** 2012-11-30

**Authors:** Hye Kyung Kim, Ji Young Lee, Hyo-Sang Han, Young-Jin Kim, Hyun Joo Kim, Yoon-Sang Kim, Hyung Min Kim, Seong-Gyu Ko, Hyo-Jin An, Young-Jong Lee, Wansu Park

**Affiliations:** 1 College of Korean Medicine, Gachon University, Seongnam 461-701, Korea; Email: doin303@hanmail.net (H.K.K.); oxygen1119@hanmail.net (J.Y.L.); godsentry@naver.com (Y.-J.K.); eternity0304@daum.net (H.J.K.); komy@Gachon.ac.kr (Y.-S.K.); garak@Gachon.ac.kr (Y.-J.L.); 2 College of Social Science, Joongbu University, Geumsan 312-702, Korea; Email: hanhs@joongbu.ac.kr; 3 College of Oriental Medicine, Kyung Hee University, Seoul 130-701, Korea; Email: hmkim@khu.ac.kr (H.M.K.); epiko@khu.ac.kr (S.-G.K.); 4 College of Oriental Medicine, Sangji University, Wonju 220-702, Korea; Email: hjan@sj.ac.kr

**Keywords:** *Liriope platyphylla*, immunomodulatory, macrophage, cytokine, nitric oxide, transcription factor

## Abstract

The tuber of *Liriope platyphylla* Wang et Tang (Liliaceae), also known as Liriopis tuber, is famous in Oriental medicine owing to its tonic, antitussive, expectorant and anti-asthmatic properties. In the present study, the effects of Liriopis tuber water extract (LP) on proinflammatory mediators secreted from lipopolysaccharide (LPS)-induced cultured RAW 264.7 mouse macrophages were investigated. Nitric oxide (NO), prostaglandin E2 (PGE2) and intracellular calcium release were measured after 24 h incubation. Various cytokines and nuclear transcription factors (NF-κB and CREB) of LPS-induced RAW 264.7 were measured by a multiplex bead array assay based on xMAP technology. LP (up to 200 μg/mL) significantly decreased levels of nitric oxide (NO), interleukin (IL)-6, IL-10, IL-12p40, interferon-inducible protein-10, keratinocyte-derived chemokine, monocyte chemotactic protein-1, vascular endothelial growth factor, granulocyte macrophage-colony stimulating factor, platelet derived growth factor, PGE2, intracellular calcium, NF-κB and CREB in LPS-induced RAW 264.7 cells (*p* < 0.05). The results suggest that LP has immunomodulatory activity to reduce excessive immune reactions during the activation of macrophages by LPS. Further studies are needed to verify the precise mechanism regulating immunomodulatory activities of LP.

## 1. Introduction

Tubers of *Liriope platyphylla* Wang et Tang (LP; family Liliaceae), also known as Liriopis tuber, have long been used as a medicinal herbal drug in East Asia (e.g., Korea, China, and Japan) owing to its tonic, antitussive, expectorant and anti-asthmatic properties [1]. In traditional Korean medicine, LP is applied to treat a dry cough with sticky sputum, physical cough, hemoptysis, pertussis, asthma, vexation and insomnia. Active compounds, such as homoisoflavone, ophiophogonone, spicatoside and ophiopogonin, have been isolated from LP [2].

Macrophages and monocytes play important roles in the inflammatory response and serve as an essential interface between innate and adaptive immunity through release of inflammatory factors, such as nitric oxide (NO), prostaglandins and various cytokines [3].

NO plays an important role in numerous physiological responses, including blood pressure regulation, wound repair, neurotransmission and host defense mechanisms, and pathophysiological responses, including inflammation, infection, neoplastic diseases, liver cirrhosis, diabetes and neurotoxicity [4]. Cytokines and growth factors are the major orchestrators of host defense processes and, as such, are involved in responses to exogenous and endogenous insults, repairs and restoration of homeostasis [5]. But, the excessive and continuing production of NO and cytokines in response to bacterial lipopolysaccharide (LPS) or superantigens is a hallmark of the systemic inflammatory response (IR), which can be life-threatening, and dissemination of these bacterial products induces waves of proinflammatory cytokines that cause vascular injury and multiple organ dysfunction [6].

Because different cytokines possess biologically overlapping functions and have the ability to regulate production of other cytokines, analysis of the function of the complete set of cytokines expressed within microenvironments (e.g., at the site of inflammation) is often of more value than analysis of a single isolated cytokine [7].

In the present study, the inhibitory effects LP on the production of inflammatory mediators, such as NO, interleukin (IL)-6, IL-10, IL-12p40, interferon-inducible protein (IP)-10, keratinocyte-derived chemokine (KC), vascular endothelial growth factor (VEGF), platelet derived growth factor (PDGF-BB), granulocyte macrophage colony-stimulating factor (GM-CSF), monocyte chemotactic protein (MCP)-1 and prostaglandin E2 (PGE2) were studied in LPS-induced RAW 264.7 mouse macrophages.

## 2. Materials and Methods

### 2.1. Reagents

Dulbecco’s Modified Eagle’s Medium (DMEM), heat-inactivated fetal bovine serum (FBS), penicillin and streptomycin, phosphate-buffered saline (pH 7.4), and other tissue culture reagents were purchased from Gibco BRL (Grand Island, NY, USA). LPS, 3-(4,5-dimethyl-2-thiazolyl)-2,5-diphenyltetrazolium bromide (MTT), Griess reagent and all other chemicals were purchased from Sigma-Aldrich (St. Louis, MO, USA). The multiplex bead-based cytokine assay kits used for the determination of cytokine concentration was purchased from Bio-Rad (Hercules, CA, USA) and Panomics (Redwood City, CA, USA). The PGE2 parameter assay kit was purchased from R & D Systems (Minneapolis, MN, USA). The Fluo-4 calcium assay kit was purchased from Molecular Probes (Eugene, OR, USA). The Procarta Transcription Factor assay kit for nuclear factor kappa B (NF-κB) and cyclic adenosine monophosphate response element-binding protein (CREB) was purchased from Panomics.

### 2.2. LP Preparation

The commercial product of *L. platyphylla *was purchased from Omniherb Company (Daegu, Korea). A voucher specimen (No. 2008-10-0012) was deposited at the College of Korean Medicine, Kyungwon University Herbarium. Because it is traditionally extracted using water in Oriental medicine, *L. platyphylla *(50 g) was extracted with 2 L of boiling water for 2 h, filtered and then lyophilized, producing an average yield of 30.2%. The powdered extract (LP) was dissolved in saline and then filtered through a 0.22 μm syringe filter.

### 2.3. Cell Culture and Viability

RAW 264.7 mouse macrophages were obtained from the Korea Cell Line Bank (Seoul, Korea). Cells were cultured in DMEM supplemented with 10% FBS containing 100 U/mL of penicillin and 100 μg/mL of streptomycin at 37 °C in a 5% CO_2_ humidified incubator. Cell viability was assessed using MTT assay.

### 2.4. Quantification of NO Production

After RAW 264.7 cells (2 × 10^4^ cells/well) were seeded in wells of a 96-well plate, LPS (1 μg/mL) and LP were added to culture medium, and incubation was continued for 16 or 24 h at 37 °C. The supernatants were collected from each well, and NO concentration was determined via the Griess reaction. Specifically, 100 μL of supernatant from each well was mixed with 100 μL of Griess reagent in wells of a separate 96-well plate. After 15 min incubation at room temperature, the optical density was determined at 540 nm with a microplate reader.

### 2.5. Multiplex Bead-Based Cytokine and Growth Factor Assay

After RAW 264.7 cells were seeded in wells of a 96-well plate, LPS (1 μg/mL) and LP were added to the culture medium, and incubation was continued for 24 h at 37 °C. The supernatant was collected from each well, and cytokines were measured using a Luminex assay based on xMAP technology. This assay was performed with Bio-Plex cytokine assay kits (Bio-Rad), Procarta cytokine assay kits (Panomics) and Bio-Plex 200 suspension array system (Bio-Rad) as described previously [8,9,10,11,12]. Standard curves for each cytokine were generated using the kit-supplied reference cytokine samples. Tested cytokines were IL-2, IL-3, IL-5, IL-6, IL-10, IL-12p40, IL-17, IL-18, IP-10, KC, VEGF, MCP-1, GM-CSF, PDGF-BB, granulocyte colony-stimulating factor (G-CSF) and basic fibroblast growth factor (basic-FGF).

### 2.6. PGE2 Assay

After RAW 264.7 cells were seeded in wells of a 96-well plate, LPS (1 μg/mL) and LP were added to the culture medium, and incubation was continued for 24 h at 37 °C. The supernatant was collected from each well and PGE2 levels were determined using a PGE2 parameter assay kit.

### 2.7. Intracellular Calcium Assay

After RAW 264.7 cells were seeded in wells of 96-well plates, LPS and LP were added to the culture medium and incubation was carried out for 30 min at 37 °C. Thereafter, the medium was removed, and cells were incubated with 100 μL of the Fluo-4 dye loading solution for 30 min at 37 °C. After incubation, the fluorescence intensity of each well was determined spectrofluorometrically (Dynex) with excitation and emission filters of 485 nm and 535 nm, respectively [10,11,12].

### 2.8. Multiplex Bead-Based Transcription Factor Assay

After RAW 264.7 cells were incubated with LPS (1 μg/mL) and LP for 4 h, nuclear extracts were prepared from RAW 264.7 cells. Nuclear localized NF-κB and CREB were quantified using a Procarta Transcription Factor Plex assay kit based on xMAP technology [13]. All reagents required for preparing nuclear extracts and performing a transcription factor assay were included and used according to manufacturer’s instructions.

### 2.9. Statistical Analysis

The results shown were from three independent experiments and represent the mean ± SEM. Significant differences were examined using Student’s *t*-test with SPSS 11.0 software (SPSS, Chicago, IL, USA).

## 3. Results

**Figure 1:**
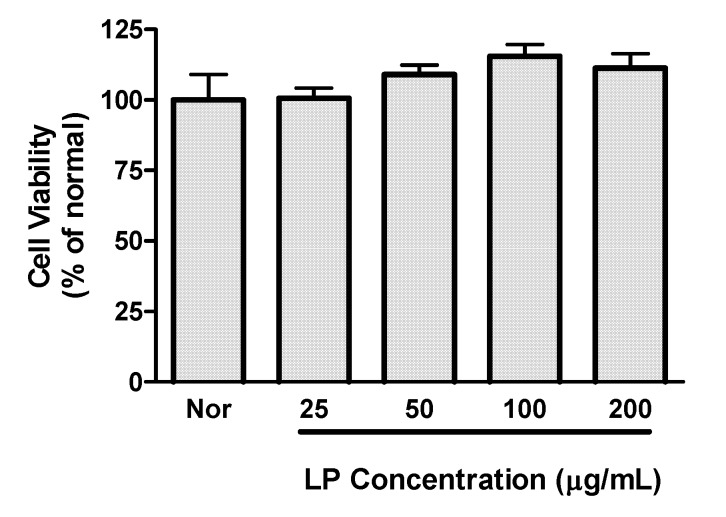
Effects of LP on cell viability. Cell viability was evaluated by a MTT assay 24 h after LP treatment in RAW 264.7 macrophages. Values are the mean ± SEM of three independent experiments.

Preliminary experiments established that LP concentrations up to 200 μg/mL did not decrease viability of RAW 264.7 macrophages (Figure 1); concentrations of up to 200 μg/mL of LP were chosen for subsequent experiments.

**Figure 2:**
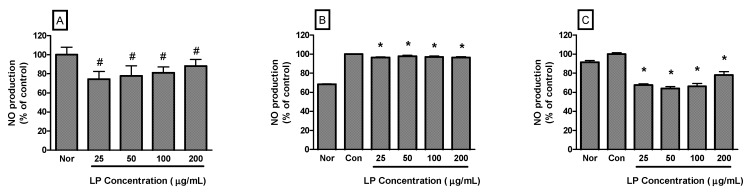
Effects of LP on NO production in RAW 264.7 macrophages for 24 h (**A**) or LPS-stimulated RAW 264.7 macrophages for 16 h (**B**) and 24 h incubation (**C**). NO production was measured by the Griess reaction assay and is expressed as a percentage of the normal (Nor; medium only) or the control (Con; LPS alone). Values are the mean ± SEM of three independent experiments. ^#^* p* < 0.05 *vs.* Nor; * *p* < 0.05 *vs.* LPS (1 μg/mL) alone.

NO production by RAW 264.7 cells incubated only with LP at concentrations of 25, 50, 100 and 200 μg/mL for 24 h were 74.37% ± 8.14%, 77.65% ± 10.82%, 81.12% ± 6.06% and 88.25% ± 6.78% of the normal (medium only) value (*p *< 0.05), respectively (Figure 2A). In the case of RAW 264.7 cells activated by LPS, NO production by RAW 264.7 cells incubated with LP at concentrations of 25, 50, 100 and 200 μg/mL for 16 h were 96.48% ± 0.55%, 97.93% ± 0.88%, 97.2% ± 0.84% and 96.58% ± 0.69% of the control (LPS only) value (*p *< 0.05), respectively (Figure 2B). For 24 h incubation, NO production by LPS-activated RAW 264.7 cells incubated with LP at concentrations of 25, 50, 100 and 200 μg/mL were 67.69% ± 1.22%, 64.02% ± 2.05%, 66.29% ± 2.91% and 78.19% ± 3.47% of the control (LPS only) value (*p *< 0.05), respectively (Figure 2C).

**Figure 3:**
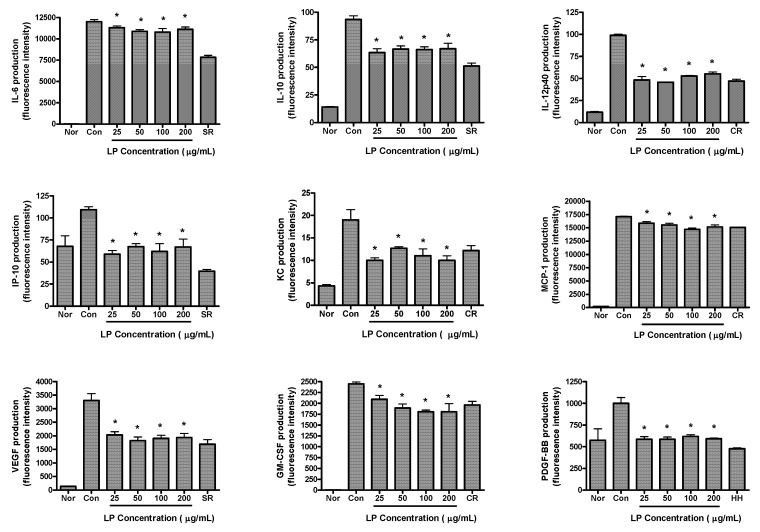
Effects of LP on cytokine production (*i.e.*, IL-6, IL-10, IL-12p40, IP-10, KC, MCP-1, VEGF, GM-CSF and PDGF-BB) in LPS-stimulated RAW 264.7 macrophages. Fluorescence intensity (FI) of various proinflammatory mediators in the culture medium was measured by Multiplex bead-based cytokine assay after 24 h incubation. Normal group (Nor) was treated with medium only. Control group (Con) was treated with LPS (1 μg/mL) alone. As reference materials, water extracts of Scutellariae Radix (SR; 100 μg/mL), Coptidis Rhizoma (CR; 12.5 μg/mL) and Houttuyniae Herba (HH; 25 μg/mL) were treated with LPS for 24 h. Values are the mean ± SEM of three independent experiments. * *p *< 0.05 *vs.* Con.

The effects of LP on production of cytokines in LPS-activated RAW 264.7 cells are shown in Figure 3. RAW 264.7 cells were treated with various concentrations of LP (25, 50, 100 and 200 μg/mL) and LPS for 24 h. LP significantly decreased the production of IL-6, IL-10, IL-12p40, IP-10, KC, VEGF, MCP-1, GM-CSF and PDGF-BB in LPS-activated mouse macrophages (*p *< 0.05 *vs.* LPS alone).

**Figure 4:**
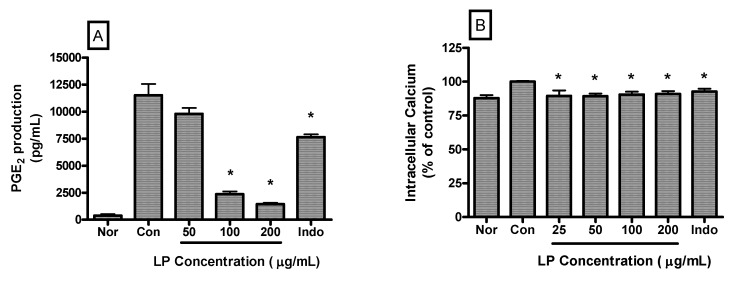
Effects of LP on PGE2 production (**A**) and calcium release (**B**) in LPS-stimulated RAW 264.7 macrophages. PGE2 concentrations in the culture medium were measured after 24 h incubation by a PGE2 parameter assay. And, intracellular calcium release was measured after 30 min incubation by Fluo-4 assay. Normal group (Nor) was treated with medium only. Control group (Con) was treated with LPS (1 μg/mL) alone. Indo denotes indomethacin (0.5 μM) with LPS. Values are the mean ± SEM of three independent experiments. * *p *< 0.05 *vs.* Con.

PGE2 production by LPS-induced RAW 264.7 cells incubated with LP at concentrations of 50, 100 and 200 μg/mL for 24 h were 9793.00 ± 544.80, 2365.00 ± 253.20 and 1446.00 ± 125.80 pg/mL, respectively. The value of the normal (medium only) and the control (LPS only) were 394.33 ± 142.80 and 11516.80 ± 1025.00 pg/mL, respectively (Figure 4A). LP significantly decreased PGE2 production in LPS-activated mouse macrophages at a concentration of 100 and 200 μg/mL (*p *< 0.05 *vs.* LPS alone).

LP also decreased the intracellular calcium release in LPS-induced RAW 264.7 macrophages significantly (*p *< 0.05). Intracellular calcium releases in LPS-induced RAW 264.7 cells incubated with LP at concentrations of 25, 50, 100 and 200 μg/mL for 30 min were 89.5% ± 4.06%, 89.24% ± 1.98%, 90.58% ± 2.12%, and 90.91% ± 2.13% of the control (1 μg/mL of LPS alone) value, respectively (Figure 4B).

**Figure 5:**
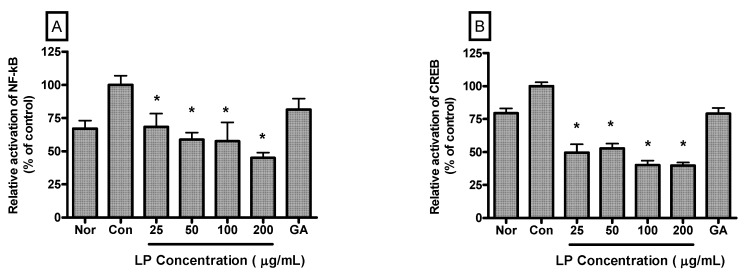
Effects of LP on NF-κB (**A**) and CREB (**B**) activation in LPS-stimulated RAW 264.7 macrophages. Nuclear localized NF-κB and CREB were quantified using a Procarta Transcription Factor Plex assay after 4 h incubation. Normal group (Nor) was treated with medium only. Control group (Con) was treated with LPS (1 μg /mL) alone. As a reference material, gallic acid (GA; 100 μM), one of important anti-oxidative and anti-inflammatory compounds, was treated with LPS for 4 h. Values are the mean ± SEM of three independent experiments. * *p *< 0.05 *vs.* Con.

Relative activation of NF-κB in LPS-induced RAW 264.7 cells incubated with LP at concentrations of 25, 50, 100 and 200 μg/mL for 4 h were 68.41% ± 10.1%, 58.79% ± 5.29%, 57.55% ± 14.22% and 45.09% ± 3.77% (*p* < 0.05) of the control (1 μg/mL of LPS alone) value, respectively (Figure 5A). And relative activation of CREB in LPS-induced RAW 264.7 cells incubated with LP at concentrations of 25, 50, 100 and 200 μg/mL for 4 h were 49.57% ± 6.27%, 52.74% ± 3.54%, 40.1% ± 3.36% and 39.82% ± 2.29% (*p* < 0.05) of the control (1 μg/mL of LPS alone) value, respectively (Figure 5B).

## 4. Discussion

For various acute and chronic inflammatory diseases, including autoimmune and allergic reaction, more effective and safe treatments are still needed. Herbal medicine, especially traditional oriental medicines long used in Korea and China, may be beneficial candidates for the alleviation of inflammatory and immune diseases. Although an inhibitory effect of LP on ovalbumin-induced airway inflammation and bronchial hyperresponsiveness has been reported in a murine model of asthma [14], the effects of LP on proinflammatory mediators secreted from inflammatory leukocytes, such as macrophages have remained unclear.

The LPS endotoxin derived from the outer membrane of gram-negative bacteria activates monocytes and macrophages to produce proinflammatory cytokines, such as IL-1, IL-6, IL-8, IL-12 and tumor necrosis factor-a [15]. In the current study, the anti-inflammatory effects of LP were investigated using RAW 264.7 mouse macrophages, stimulated with LPS.

There is ample evidence for the occurrence of inflammatory processes, which include activation of microglia (the resident macrophages of the brain and spinal cord) and astrocytes (star-shaped glial cells), with subsequent release of cytokines and other inflammatory factors, such as NO, in most major neurodegenerative disorders, both in acute conditions, such as traumatic brain injury and stroke, and in chronic disorders, such as Alzheimer’s disease, epilepsy, amyotrophic lateral sclerosis and Parkinson’s disease [16]. During exacerbations of multiple sclerosis, elevated levels of IP-10 in cerebrospinal fluid affect T cells and mononuclear phagocytes [17]. The present study found inhibitory effects of LP on LPS-induced NO and IP-10 production in RAW 264.7 macrophages. These results suggest that LP may be a candidate to counteract neurodegenerative brain inflammation by targeting the production and release of proinflammatory molecules, such as NO and IP-10.

IL-10, although traditionally considered an anti-inflammatory cytokine, has also been implicated in promoting abnormal angiogenesis in the eye and in the pathobiology of autoimmune diseases, such as lupus and encephalomyelitis [18]. IL-6, IL-12 and VEGF are involved in the development of endometriosis with excessive endometrial angiogenesis [19], and overexpressions of VEGF and PDGF have been linked to different types of malignancies and tumors [20]. In the present study, LP showed an inhibitory effect on production of IL-6, IL-10, IL-12p40, VEGF and PDGF-BB in RAW 264.7 macrophages stimulated by LPS. Thus, it can be suggested that LP is a candidate for treatment of various diseases concerned with inflammatory angiogenesis such as endometriosis, lupus, encephalomyelitis and tumors. 

GM-CSF plays an important role in high-dose LPS- and hemorrhage-induced acute lung injury (ALI), which appears to be mediated by its priming effect on neutrophils [21]. KC and MCP-1 expression are increased within lung homogenate from the mouse of bacterial pneumonia [22], and PGE2 enhances fibroblast proliferation, which results in severe, persistent respiratory dysfunction in ALI [23]. With the inhibitory effect of LP on excessive production of GM-CSF, KC, MCP-1 and PGE2 in LPS-induced RAW 264.7 cells, LP may relieve pulmonary inflammatory disease, such as ALI and bronchial pneumonia.

NF-κB and CREB, important transcription factors (TFs) in inflammation, are critical activators in the expression of proinflammatory proteins, such as NOS, cytokines and COX in macrophages [24,25]. The presently observed inhibitory effect of LP on LPS-induced activation of NF-κB and CREB in RAW 264.7 cells suggests that LP might inhibit production of inflammatory mediators in LPS-induced macrophages via suppression of transcriptional activators, such as NF-κB and CREB.

LPS-induced intracellular calcium in macrophages promotes NF-κB and ERK 1/2 activation, which are major pathway of Toll-like receptor, signaling into production of inflammatory mediators, including NO and cytokines [26]. In the present study, LP inhibited LPS-induced calcium release in RAW 264.7 cells. Thus, inhibitory effects of LP on production of inflammatory mediators in LPS-induced RAW 264.7 cells might be achieved via regulation of the calcium-TF pathway.

As well, LP diminished the production of some cytokines, such as IL-3, IL-5, G-CSF and basic FGF in LPS-induced RAW 264.7 cells (data not shown). But, LP did not show any significant effect on the production of IL-2, IL-17 and IL-18 in LPS-induced RAW 264.7 cells.

## 5. Conclusions

Although the precise mechanisms regulating the anti-inflammatory activity of LP are not yet known, the present study has demonstrated that LP inhibits LPS-stimulated production of inflammatory mediators, including NO, IL-6, IL-10, IL-12p40, IP-10, KC, VEGF, PDGF-BB, GM-CSF, MCP-1 and PGE2 in LPS-induced RAW 264.7 mouse macrophages via regulation of the calcium-TF pathway. These findings suggest that LP possesses anti-inflammatory properties and may modulate macrophage-mediated inflammatory stimulation. Further studies are needed to verify the precise mechanism regulating anti-inflammatory activities of LP.
